# Ohio State Medical Convention

**Published:** 1849-08

**Authors:** 


*Ohio State Medical Convention.*

The annual sittings of this body were held in the Senate Chamber in this city, commencing on the 5th, and terminating on the 7th of June. A larger number of members were in attendance than at any previous meating of the Convention—120 Physicians, representing the various sections of the State, came up to exchange friendly greetings with the acquaintances of former years, to become personally acquainted with professional brethren whom they had long known by reputation, and to labor together for the advancement of Medical Science and the common good of the profession. The proceedings throughout, were characterized by great harmony. We have only room for a brief abstract, and refer our readers for a more extended notice to the published proceedings, and to the excellent reports of the "Ohio State Journal" and "Tribune."

The officers of the Convention were

*President,*

DR. PLINY M. CRUME, of Preble county.

*Vice Presidents,*

1st. Dr. E. H. Davis, of Ross county ;
2d. " J. C. Norton, of Marion ;
3d. " C. Faulkner, of Butler ;
4th. " E. Carney, of Delaware ;
5th. " W. W. Rickey, of Sandusky.

*Secretaries,*

Dr. Norman Gay, of Franklin ;
" M. Thompson, of Knox.

*Treasurer,*

Dr. J. B. Thompson, of Franklin.

A number of papers on various and interesting subjects in Medicine and Surgery, were read by members of the Convention. One of these, by Prof. Kirtland, will be found in the present No. of the Journal—others we will endeavor, from time to time to lay before our readers. They will all be found in the official report. The following resolutions in reference to extending the lecture term of our Medtcal Schools, were reported back to the convention by the committee to whom they were last year referred, and were adopted.

"*Resolved,* That in the opinion of this Convention, it is inexpedient for the Medical schools of Ohio, at this time, to lengthen the term of their sessions ; but it should be strictly enjoined upon students to be present at the beginning, and to continue in attendance until the end of the session.

*Resolved,* That the practice of admitting young men to graduate, by attending upon one course of lectures only, considering four years' practice as equivalent to to attendance upon another, is unjust to our medical Schools, and to the community, as it indirectly holds out inducements to young men to engage in the practice of medicine, without due qualifications.

2. *Resolved*, That the Professors in the Medical Schools in this State, be requested to confer together upon the subject, with a view of correcting this evil.

The Committee to whom was referred the subject of adulteration of Drugs, submitted the following report which was adopted :

WHEREAS, the extensive and multiplied means of deteriorating medical agents, as practised by wholesale dealers in drugs, abroad and at home, has now been brought before the public by the faithful and persevering labors of our *Colleges of Pharmacy* and others, whose employment has brought them into intimate relation with the subjects, and whereas the evil has been corrected in part by National Legislation, through the wise councils and untiring zeal of one of our own Representatives in Congress, and whereas the laws of Congress will ultimately be of little avail, unless sustained by the co-operation of State Legislation, and inasmuch as this can be effected only by those means which will command the assent and united efforts of all our profession ;—Therefore,

*Resolved*, That as a profession, we appeal with united voice to those engaged in the domestic drug trade, to supply us with articles of the purest character ; and that we pledge ourselves to sustain them in the effort.

*Resolved*, That we pledge ourselves to each other, and the public, that we will purchase no drugs, or chemicals, that are not pure, so far as we can judge ; and further, that we will deal with no trader whom we know to keep adulterated or impure articles.

*Resolved*, That the Convention appoint a committee of five to present to the next Legislature the crying nature of this evil, and to memorialize them, in the name of this Convention, to pass such laws as shall best sustain National Legislation on this subject.

*Resolved*, That the same committee further memorialize the Legislature on the subject of *Patent* and secret medicines, for the passage of a law requiring that all such articles sold as medicines, shall have upon them a label, in the English language, setting forth the ingredients, and the proportion of each ingredient contained in the compound.

*Resolved*, That the thanks of this Convention are due to our Representative, Dr. T. O. Edwards, for his faithful and successful efforts in the cause of pure medicines.

Profs. Smith, Kirtland, Judkins, Drs. Rickey and Gaston constitute the Committee contemplated in the third resolution.

The nature and treatment of Asiatic Cholera occupied a prominent place in the discussions of the Convention. The subjoined remarks by Prof. Judkins, of Starling Medical College, for the report of which, we are indebted to the O. S. Journal, are concise and to the point.

"Dr. JUDKINS said that it was not his intention to enter into a discussion upon the nature of Cholera, but merely to give a brief statement of his experience in the treatment of that disease. But as some remarks had been made upon the question of its being contagious or non-contagions in its character—he would say, that he was fully wedded to the belief that it was not contagious. In all of his practice he had never seen more than one case of decided cholera to occur in the same family, although many instances were known where such had been the fact.

The scourge in Cincinnati paid no respect to age or sex. Children were as liable to be attacked as adults. He had seen a child of seven months old die of cholera.

The disease is always preceded by some mild symptoms, which should be immediately attended to. One single case occurred in his practice where it was denied that any premonitory symptoms had been present.

Whilst the epidemic was at its height, but few of the inhabitants escaped some of the premonitory symptoms, such as pain in the bowels, impaired appetite, a sense of fatigue, and general uneasiness, &c. This state of things prevailed almost universally throughout the city—and it has doubtless given rise in our city to what may be called a homœopathic variety of cholera, of which several hundred cases have been reported; of that number not a single death occurred. These reports at that time, gave rise to the prediction that credulous persons would be gulled by those reports, and sooner or later the homœopathists would be called to treat cases of true cholera. Within a few days past this has been verified, and now we begin to hear of deaths from cholera under homœopathic treatment.

The general uneasiness felt by the whole population, it is believed, was caused by the want of a due proportion of oxygen in the atmosphere, a fact which appears to be established by some of our most eminent manufacturing chemists, and prominent among those I will mention Mr. Grasseli, who has one of the largest chemical Laboratories in the country—for several days he was unable to make but little over one-half of the usual amount of Sulphuric Acid.

Dr. J. observed a remarkable fact in all the cases which he saw, that terminated fatally; there was an entire absence of the cramps or spasms of the muscles, from the onset of the disease. He had lost but three cases by cholera, and no spasms were manifest in any one of them. He feels more confident of success if called to a case where cramp is felt, &c.

In the treatment of the disease, he would say that all of the regular profession in his city look upon it as easily managed, if taken early, as the ordinary attacks of any violent disease.

They all deem it of the highest importance to arrest any form of diarrhœa as speedily as possible, and one of the first things enjoined upon a patient is rest and the recumbent posture, until the diarrhœa is checked.

Frequently, before the diarrhœa has set in, the man complains of uneasiness, &c. If the tongue is large, flabby, and covered with a white fur, a blue pill given at bed time, and followed in the morning by a dose of magnesia and spiced syrup of rhubarb, to act gently on the bowels may suffice; but if he is still languid, an infusion of gentian, or something analagous, will in most cases restore the tone of the stomach and bowels. Or the same effect in other instances is produced by the spiced burnt brandy.

When called to cases laboring under diarrhœa, before vomiting has occurred, he gives to an adult, at every stool, one-half of a tablespoonful of the following mixture:

R—Chalk mixture, 2 ounces,
Tr. Kino, 1 do.
Tr. Capsicum, 1 drachm,
Elix. Paregoric, 1½ ounce.

If the stools still continue, or begin to assume a light color, give with the above at every stool, the following pills:

| | | |
|---|---|---|
| R — Calomel, | 2 | grains. |
| Camphor, | 1 | do. |
| Capsicum, | 1 | do. |
| Pulv. Opium, | ½ | do. |

Continue this until the consistence and color of the discharges are changed. He observed, in two cases, that the stools assumed a natural consistence, but still retained their light color, resembling very much the fire-clay. In these cases, small doses of blue mass, or calomel were given for several days, until healthy evacuations were procured.

When called to a case where rice water discharges were profuse, both from bowels and stomach, skin cold, vomiting almost incessant, &c., he first gave a tablespoonful of ground mustard, with a teaspoonful of common salt. After being rejected the stomach was more quiet, and the heat of the surface increased; he then began immediately with the above treatment, frequently giving the ingredients of the pill in the form of a powder. Also, applied mustard to the extremeties and over the abdomen — placed from six to ten bottles filled with hot water, covered with flannel, in contact with the body — and over all, placed an additional blanket. If a warm and profuse perspiration can be induced and maintained for several hours, his chance for recovery is good.

When the patient was verging into a stage of collapse, and the stomach so irritable as to reject all medicine and brandy, success was frequently secured by the use of the following mixture, given in teaspoonful doses every 15 or 20 minutes:

| | | |
|---|---|---|
| R—Camphor, | 1 | drachm, |
| Chloroform, | 1 | do. |
| Pep'mint water, | 3 | ounces, |
| Tr. Kino, | 1 | do. |

To allay intense thirst, very small pieces of ice were given frequently to the patient.

Also endeavored to inspire a feeling of confidence in the patient that he would recover.

Fear, grief, and other depressing emotions, were the strongest predisposing causes observed in his practice."

Remarks were also made by Profs. Mussey, Kirtland, Dr. McElvain, and others, but the absence of any reports, and want of room, prevent their insertion.

Twelve delegates to the next meeting of the National Medical Association at Cincinnati, in May next, were appointed by the Convention.

Convention adjourned to meet in Columbus on the first Tuesday in June next. Success to the next, and all subsequent meetings say we. Such assemblages for mutual consultation and encouragement must in every way, be productive of good results.—*Ohio Medical and Surgical Journal.*

